# Quorum-sensing regulates biofilm formation in *Vibrio scophthalmi*

**DOI:** 10.1186/1471-2180-12-287

**Published:** 2012-12-03

**Authors:** Cristina García-Aljaro, Silvia Melado-Rovira, Debra L Milton, Anicet R Blanch

**Affiliations:** 1 Departament de Microbiologia, Facultat de Biologia, Universitat de Barcelona, Barcelona, 08028, Spain; 2 Department of Molecular Biology, Umeå University, Umeå, 90187, Sweden

**Keywords:** Vibrio scophthalmi, Biofilm formation, Quorum-sensing, AiiA, LuxS, Acyl homoserine lactone

## Abstract

**Background:**

In a previous study, we demonstrated that *Vibrio scophthalmi*, the most abundant *Vibrio* species among the marine aerobic or facultatively anaerobic bacteria inhabiting the intestinal tract of healthy cultured turbot (*Scophthalmus maximus*), contains at least two quorum-sensing circuits involving two types of signal molecules (a 3-hydroxy-dodecanoyl-homoserine lactone and the universal autoinducer 2 encoded by *luxS*). The purpose of this study was to investigate the functions regulated by these quorum sensing circuits in this vibrio by constructing mutants for the genes involved in these circuits.

**Results:**

The presence of a homologue to the *Vibrio harveyi luxR* gene encoding a main transcriptional regulator, whose expression is modulated by quorum–sensing signal molecules in other vibrios, was detected and sequenced. The *V. scophthalmi* LuxR protein displayed a maximum amino acid identity of 82% with SmcR, the LuxR homologue found in *Vibrio vulnificus*. *luxR* and *luxS* null mutants were constructed and their phenotype analysed. Both mutants displayed reduced biofilm formation *in vitro* as well as differences in membrane protein expression by mass-spectrometry analysis. Additionally, a recombinant strain of *V. scophthalmi* carrying the lactonase AiiA from *Bacillus cereus*, which causes hydrolysis of acyl homoserine lactones, was included in the study.

**Conclusions:**

*V. scophthalmi* shares two quorum sensing circuits, including the main transcriptional regulator *luxR*, with some pathogenic vibrios such as *V. harveyi* and *V. anguillarum*. However, contrary to these pathogenic vibrios no virulence factors (such as protease production) were found to be quorum sensing regulated in this bacterium. Noteworthy, biofilm formation was altered in *luxS* and *luxR* mutants. In these mutants a different expression profile of membrane proteins were observed with respect to the wild type strain suggesting that quorum sensing could play a role in the regulation of the adhesion mechanisms of this bacterium.

## Background

*V. scophthalmi* is the most abundant species among the marine aerobic or facultatively anaerobic bacteria present in the intestinal tract of cultured turbot (*Scophthalmus maximus*) even though it is not the most abundant *Vibrio* species in the surrounding water [1,2]. However, the possible benefits of turbot colonization by this bacterium are not well understood.

Bacteria communicate with members of their own species and even with bacteria outside of the species boundary to coordinate their behaviour in response to the density of the bacterial population, which is known as quorum-sensing [3]. This communication relies on the production and sensing of one or more secreted low-molecular-mass signalling molecules, such as N-acylhomoserine lactones (AHLs), the extracellular concentration of which is related to the population density of the producing organism. Once the signalling molecule has reached a critical concentration, the quorum-sensing regulon is activated and the bacteria elicit a particular response as a population.

The first quorum-sensing system identified was shown to control bioluminescence in *Vibrio fischeri* through the LuxI-LuxR system [4,5]. LuxI synthesizes a diffusible signal molecule, N-(3-oxohexanoyl)-L-homoserine lactone (3-oxo-C6-HSL), which increases in concentration as the cell density increases. LuxR, the transcriptional activator of the bioluminescence *lux* operon, binds 3-oxo-C6-HSL, which increases its stability. This complex binds the promoter of the *lux* operon activating the production of light. The LuxI-LuxR quorum-sensing circuit is found in many Gram-negative bacteria and has been shown to regulate a variety of genes; for instance, it has been shown to regulate virulence in *Pseudomonas aeruginosa*[6]. However, this quorum-sensing circuit initially described in *V. fischeri* is not present in all *Vibrio* spp.

In *Vibrio harveyi* three additional quorum-sensing circuits were characterized that respond to three different signal molecules (see [7], for review). The first quorum-sensing system is composed of an AHL synthase, LuxM, which is responsible for the synthesis of 3-hydroxy-C4-HSL, and the receptor LuxN, a hybrid sensor kinase (present in *V. harveyi, Vibrio anguillarum* and *Vibrio parahaemolyticus*, among others). The second is composed of LuxS, LuxP and LuxQ. LuxS is responsible for the synthesis of the autoinducer 2 (AI-2), a universal signaling molecule used both by Gram-negative and Gram-positive bacteria for interspecies communication [8], LuxP is a periplasmic protein that binds AI-2 and LuxQ is a hybrid sensor kinase. The third system is composed of CqsA and CqsS. CqsA is responsible for the synthesis of a different autoinducer, the cholerae autoinducer CAI-I [9], and CqsS is the hybrid sensor kinase*.* These three quorum-sensing systems converge via phosphorelay signal transduction to a single regulator LuxO, which is activated upon phosphorylation at low cell density. LuxR, a regulatory protein that shares no homology to the *V. fischeri* LuxR, activates bioluminescence, biofilm formation, and metalloprotease and siderophore production at high cell density, is at the end of this cascade [10]. This regulatory protein is repressed at low cell density and derepressed at high cell density in the presence of autoinducers which, after binding, activate the phosphatase activity of the sensor kinases. This more complex quorum-sensing system is found predominately in *Vibrio* species and components of the network vary between species [7].

In a previous work, we demonstrated the presence of two quorum-sensing signal molecules in the supernatants of *V. scophthalmi*: *N*-(3-hydroxydodecanoyl)-L-homoserine lactone (3-hydroxy-C12-HSL) and AI-2, encoded by a *luxS* gene [11]. However, there is still a lack of knowledge of the bacterial activities that are regulated by quorum-sensing in this bacterium. In this study, we identified a homologue of the *V. harveyi luxR* transcriptional regulator and analyzed the functions regulated by LuxR and the previously identified quorum-sensing signaling molecules by constructing mutants for the coding genes.

## Results and discussion

### Detection and sequencing of *luxR* homologue

In a previous study we demonstrated the presence of two quorum sensing signals in the supernatants of *V. scophthalmi,* a 3-hydroxy-C12-HSL and the AI-2 [11]. This fact suggested that *V. scophthalmi* could have two quorum-sensing circuits homologous to those identified in *V. harveyi* that converge in the *luxR* transcriptional regulator. In the present study the genome of *V. scophthalmi* A089 and A102 strains was screened by PCR analysis for the presence of *luxR* homologues using the primers listed in Table 1. For *luxR*, a 636-bp fragment was generated and sequence analysis showed that this fragment shared high similarity to the *V. harveyi*-like *luxR* transcriptional regulator, which belongs to the TetR subfamily of transcriptional regulators [12]. The sequence of the complete *luxR* gene obtained by inverted PCR and showed a maximum nucleotide identity with *V. parahaemolyticus* (75%) although the maximum amino acid identity and similarity was with *V. vulnificus* (82% and 90%, respectively) (Table 2). In addition, the 5’- and 3’-flanking DNA sequence of the *luxR* gene was also determined. The upstream region showed 87% identity with an intergenic region of *V. tubiashii* located between the hypoxanthine phosphoribosyltransferase (*hpt*) gene and *luxR*[13]. The downstream region of the *V. scophthalmi luxR* gene contained an ORF that showed a maximum identity of 87% with the dihydrolipoamide dehydrogenase gene (*lpd*) of *V. parahaemolyticus*[14]. This genetic organization has also been described in some other vibrios such as *V. cholerae* and *V. vulnificus*[15], suggesting that they have been acquired by vertical transmission from a common ancestor.

**Table 1:** Primers used in this study

	**Sequence (5’-3’)**	**Target gene**	**Reference**
LuxR-A	GG**ACTAGT**TACTAATTAGGGCAA	*luxR* null mutant	This study
LuxR-B	ATAAATACACAACATGAGTCGGGTGCGGGG	*“*	*“*
LuxR-C	ATGTTGTGTATTTATAAAGAAGAA	*“*	*“*
LuxR-D	CTC**GAGCT**CGAGTCAGTGGGTCTA	*“*	*“*
LuxR-G	CCG**GAATTC**CATTTGGCAAGGATT	Over expression *luxR*	*“*
LuxR-H	CGC**GGATCC**GGTGATGAGTTTCAC	*“*	*“*
LuxR-1	CCATTACACTCATAAGCGCGA	Sequencing *luxR* and	*“*
LuxR-2	TCGAGATGGGTTGTGACGCTG	flanking regions	*“*
LuxRI-F2	GCACCATTACACTCAT	Detection of *luxR*	*“*
LuxRI-R2	TTTGATGAACATGTTTTG	*“*	*“*
LuxRI-F4	AAGTGTGGTTTGAGTGGA	Detection of *luxR* and	*“*
LuxRI-R4	TAAGCAACAGCTGATGGA	flanking regions	*“*
LuxS-F6	CGATCTTGCTCTACCGGCT	Sequencing *luxS*	*“*
LuxS-R7	GAGTGCATCGCTGCAGTAC	flanking regions	*“*
LuxS-A	GG**ACTAGT**CTGGCTTATCACGAAG	*luxS* null mutant	*“*
LuxS-B	CTCATTGAGCATTCGACAGTAAAGCTATC	*“*	*“*
LuxS-C	GAAATGCTCAATGAGCTTCGCGTC	*“*	*“*
LuxS-D	CTC**GAGCT**CGGACACTCGATCCACA	*“*	*“*
LuxS-PMMBF	CCG**GAATTC**GCCAGCAGGAGAAGGACA	Over expression *luxS*	*“*
LuxS-PMMBR	CGC**GGATCC**CGCTATCGATTAATCGA	*“*	*“*
LuxS-AI	**GGATCC**GCCAGCAGGAGAAGGACA	Cloning of *luxS* into pACYC184	*“*
LuxS-BI	**GTCGAC**CGCTATCGATTAATCGAC		*“*

Restriction sites for *Spe*I (ACTAGT), *Bam*HI (GGATCC), *Eco*RI (GAATTC), *Sal*I (GTCGAC) and *Sac*I (GAGCT) are indicated in bold.

**Table 2:** **Percentage of nucleotide and amino acid identity and similarity of*V. scophthalmi*A089 LuxR with previously reported*V. harveyi*-like LuxR regulators**

**Species**	**% nt id (% aa id/% aa sim)**
*V. alginolyticus* (AF204737.1)	74% (81%/90%)
*V. anguillarum* (AF457643.2)	73% (80%/89%)
*V. cholerae* (EU523726.1)	73% (76%/87%)
*V. harveyi* (M55260.1)	73% (79%/90%)
*V. mimicus* (AB539839.1)	71% (77%/86%)
*V. parahaemolyticus* (AF035967.1)	75% (80%/90%)
*V. vulnificus* (EF596781.1)	75% (82%/90%)

GenBank Accession Number in brackets; nt, nucleotide; aa, amino acid; id, identity; sim, similarity.

### Functions regulated by *luxR*, *luxS* and AHLs

In order to uncover the functions regulated by quorum-sensing in *V. scophthalmi* null mutants for *luxR* and *luxS* were constructed. Additionally, a recombinant strain generated in a previous study that carries a gene coding for a lactonase from *Bacillus cereus* (AiiA) which was previously shown to hydrolyse AHLs [11] was included in the assays to study the functions regulated by AHLs.

**Figure 1:**
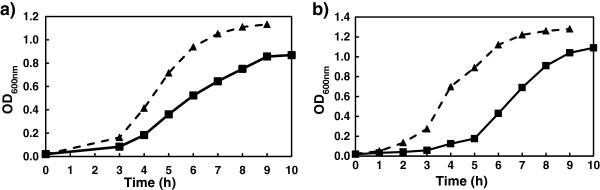
**a) Effect of overexpression of*luxR*on the growth rate of*V. scophthalmi*.*V. scophthalmi*A089_23 (pMMB207)*(black triangle)*used as control strain*vs V. scophthalmi*A089_23 (pMMB207*::*luxR)*(black square)*; b) Effect of expression of the lactonase*aiiA*in*V. scophthalmi*A102.** The growth rate was reduced in *V. scophthalmi* A089_23 overexpressing luxR (*black square*) compared to the control strain (black triangle) (Figure 1a), while strain A102_6.2 expressing the lactonase (black square) had a longer lag phase with respect to the control strain A102_pACYC (*black triangle*) (Figure 1b).

No differences in growth rates were detected between the *luxR* and *luxS* mutants and the wild type strains. However, over-expression of *luxR* resulted in a decreased growth rate. The strains over-expressing *luxR* arrived to the stationary phase with a delay compared to the *luxR* mutant carrying the plasmid alone (Figure 1a). Similarly, although motility was not affected with statistical significance in *luxR* and *luxS* null mutants, over-expression of *luxR* caused about 50% decrease in motility in the swimming plate assay (31.8 mm ^+^/_−_ 7.6 mm in the strain over-expressing *luxR* and 54.3 mm ^+^/_−_ 8.1 in the control strain, after 24 hours), which is likely due to the decrease in the growth rate and not to downregulation of the genes involved in motility. The recombinant strain carrying the lactonase AiiA, had a much longer lag phase before reaching exponential growth which was then at a similar rate to that of the parent strain (Figure 1b) and showed also a reduction about 50% of motility with respect to the control strain (11.5 mm ^+^/_−_ 3.3 mm in the recombinant strain and 24.0 mm ^+^/_−_ 6.5 mm in the control strain). In the case of *luxS* over-expression no differences in the growth rate was observed for any of the strains.

**Figure 2:**
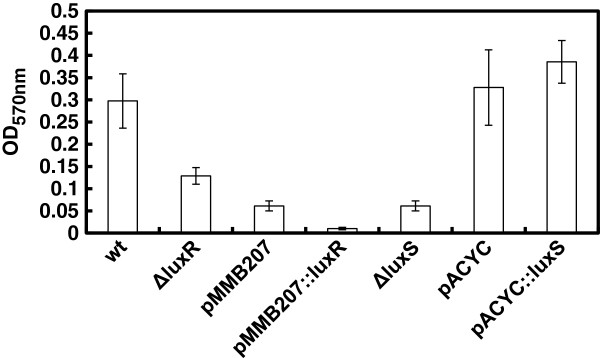
**Biofilm formation in the*V. scophthalmi*A102 strain cultured in MB; wt, wild type strain;*ΔluxR*, A102_56 strain;*ΔluxS*, mutant A102_73 strain; pMMB207, A102_90; pMMB207*::luxR*, A102_78 mutant; pACYC, A102_pACYC184; pACYC184::*luxS*, A102_99 strain.** The error bars indicate standard deviation based on three independent assays with four replicates each one. Statistical analysis was performed by student’s *t* test. Similar results were obtained with the A089 mutant strains.

In contrast, quorum-sensing was shown to positively regulate biofilm formation *in vitro* since both *luxR* and *luxS* null mutants had altered biofilm formation (Figure 2). Noticeably, biofilm was only formed when bacteria were grown in MB medium in either the mutant or the wild-type strains and abolished when bacteria were cultured in TSB2 (data not shown). MB medium is used to culture heterotrophic marine bacteria and mimics the marine salt concentration and, although TSB also allowed growth of the bacterium, for some reason the differences in salt concentration or in nutrient or carbohydrate contents exerted an effect on biofilm formation. In order to investigate a possible effect of catabolite repression, we supplemented MB with glucose 0.5% and 1% w/v which resulted in a decrease in biofilm formation. On the other hand, over-expression of *luxR* decreased the amount of biofilm, perhaps due to the decrease in the growth rate caused by the deregulation of *luxR*, as stated above. In the case of *luxS* overexpression no differences were found between the over-expressed *luxS* and the control strain carrying pMMB207 plasmid. Complementation of the A102 null *luxS* mutant strain with the pACYC184 plasmid reverted the strain to the wild type phenotype.

Positive and negative regulation of biofilm formation has been reported in other vibrio such as *V. anguillarum* and *V. cholerae,* respectively [16,17]. Interestingly, in a recent study on quorum-sensing in *V. ichthyoenteri* (the most closely related species to *V. scophthalmi*), its *luxS* homologue was sequenced and a mutant for this gene constructed, but no functions were reported to be regulated by this gene [18]. It has to be noted that neither the *V. ichthyoenteri* wild type, nor the *luxS* mutant formed biofilms in the microwell plates. Our results showed that *luxS* is involved in biofilm formation at least *in vitro* in *V. scophthalmi*. However, it is important to highlight that in our study the *V. scophthalmi* wild-type strain was only able to form significant biofilm when grown in MB, while TSB inhibited biofilm formation *in vitro*. Therefore, it would be interesting to assess if *V. ichthyoenteri* and the *luxS* mutant behave similarly to *V. scophthalmi* since they are so closely related. In *V. scophthalmi*, these two quorum-sensing systems may play a role in the colonization and establishment of this bacterium in the fish intestine, since it is a normal inhabitant of the turbot intestine [1]. In fact most vibrio species form biofilms on different structures, which is believed to be beneficial for the populations against different environmental stresses [19]. Work is currently being done to test these hypotheses.

**Figure 3:**
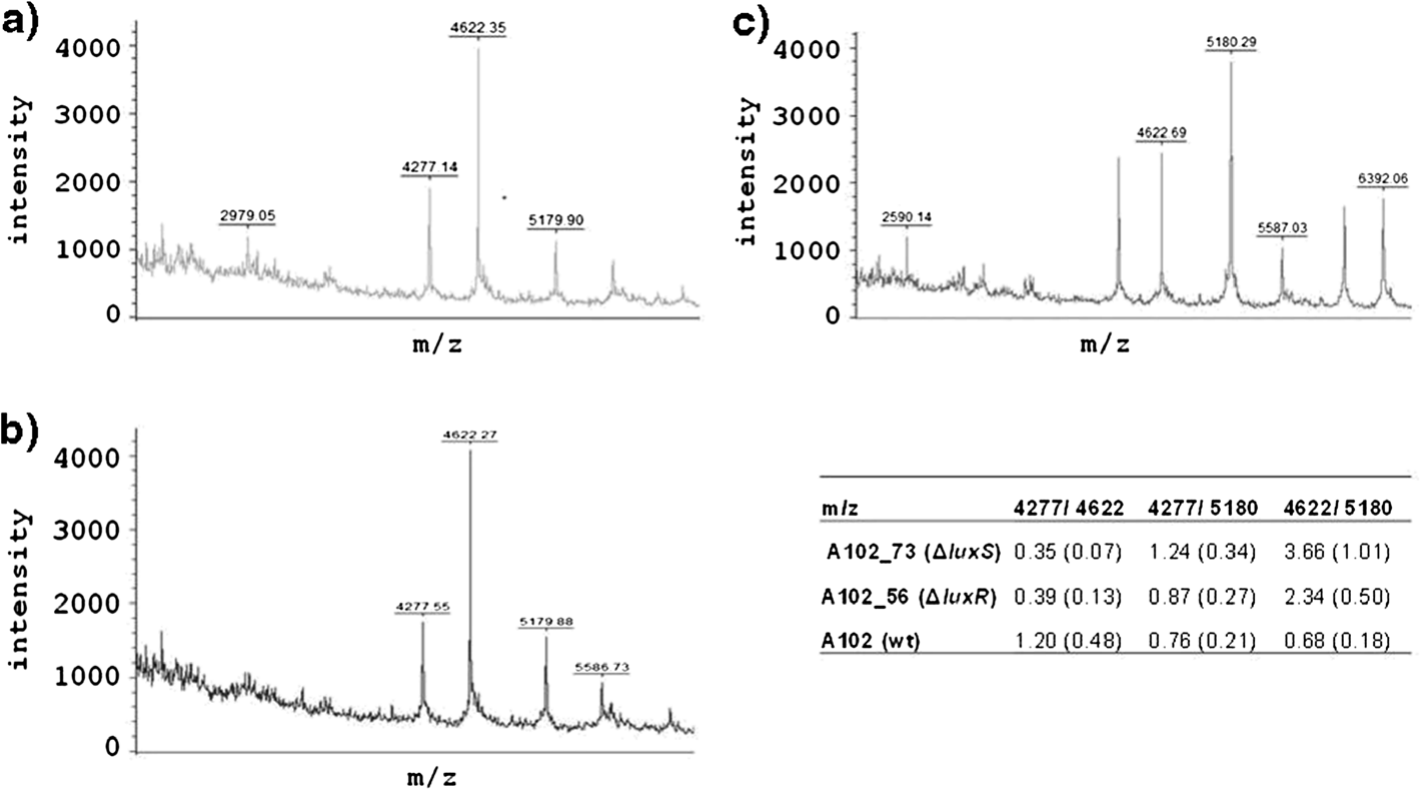
**Differential expression of membrane proteins in the (a)*V. scophthalmi*A102*luxS*and (b)*luxR*mutants with respect to the (c) wild type strain analyzed by mass spectrometry.** The ratios of the major proteins with m/z 4277, 4622, 5180 are shown in the inset. Standard deviation of three independent measurements in brackets.

A difference in the expression of membrane proteins, which may relate to differences in biofilm formation, was assessed by mass spectroscopy. In the case of the *luxS* mutant the intensity of m/z 4277 was significantly lower than m/z 4622 and m/z 4622 was significantly higher than m/z 5180, while in the wild type strain these ratios were reversed (p<0.01) (Figure 3). Similar results were obtained for the *luxR* mutant, suggesting that the expression of these proteins were affected by these mutations.

Extracellular protease activity was not detected in either the wild-type strain or any of the *luxR* and/or *luxS* mutants as determined by a qualitative milk plate assay as well as a quantitative detection method using azocasein. On the other hand, siderophore production, which has been shown to be regulated by quorum-sensing in other vibrios was evaluated using the siderophore CAS assay. In addition, the ability to grow in iron depleted medium (EDDA assay) was assessed. A minor positive signal indicating the presence of siderophore activity was detected in all the mutants and wild type strains with the same intensity. However, neither the wild-type strain nor the mutants grew in the EDDA-supplemented medium suggesting that this species is not able to grow in iron-depleted medium, at least under the conditions used in the assay. Extracellular proteases and siderophores are often produced by pathogenic vibrios [20-22], although some vibrios that are not pathogenic have been shown to produce siderophores in an iron-limited host environment, such as *V. fischeri*[23].

The *Vibrio harveyi*-like LuxR family of regulators is a diverse family with different associated functions depending on the *Vibrio* species. For example, in *V. harveyi*, *luxR* is expressed at high cell densities and regulates different functions including siderophores, colony morphology, activates bioluminescence, activates metalloprotease production, represses the type III secretion system [21,24,25]. Apart from this diversity, all the *V. harveyi*-like quorum-sensing systems converge to a phosphorelay circuit that regulates the expression of *luxR*. However, in *V. anguillarum,* contrary to other members of the LuxR family, this gene is expressed at low densities. This gene represses exopolysaccharide production, and regulates biofilm formation, metalloprotease, pigment production and serine biosynthesis [17]. In the case of *V. scophthalmi*, which is a non-pathogenic vibrio, no virulence factors are shown to be regulated by this transcriptional regulator. At this moment, genome sequencing of the two *V. scophthalmi* strains used in this study is under process in our laboratory. Future work will involve transcriptome analysis of these mutants.

## Conclusions

*V. scophthalmi* shares two quorum sensing circuits, including the main transcriptional regulator LuxR, with some pathogenic vibrios such as *V. harveyi* and *V. anguillarum*. However, contrary to these pathogenic vibrios no virulence factors (such as protease or siderophore production) were found to be quorum sensing regulated in this bacterium. Noteworthy, biofilm formation was altered in *luxS* and *luxR* mutants. In these mutants a different expression profile of membrane proteins were observed with respect to the wild type strain suggesting that quorum sensing could play a role in the adhesion and subsequent colonization of the fish by this bacterium. Further studies are needed in order to ascertain a similar behaviour of these mutants *in vivo*.

## Methods

### Bacterial strains, culture media and growth conditions

The bacterial strains and plasmids used in this study are listed in Table 3. The *V. scophthalmi* strains were grown at 30°C with agitation at 180 rpm in either marine broth (MB, Difco) (filtered through a 0.1 μm pore size to remove any precipitated salts that normally occur in this medium), or tryptic soy broth (TSB, Difco) supplemented with NaCl to a final concentration of 2% (TSB2). Luria Bertani (LB) broth was used for growth of *Escherichia coli*. When needed, antibiotics were added to the media at the following final concentrations: 5 μg/ml and 25 μg/ml chloramphenicol for *V. scophthalmi* and *E. coli*, respectively, and 100 μg/ml ampicillin for *E. coli*.

**Table 3:** Bacterial strains and plasmids used in this study

**Strain or plasmid**	**Genotype and feature(s)**	**Reference**
*V. scophthalmi* strains		
A089	Wild type, turbot isolate (CECT 4638T)	[2]
A102	Wild type, turbot isolate (CECT 5965)	[1,2]
A089_23	A089 Δ*luxR* mutant	This study
A089_88	A089_23 (pMMB207)	“
A089_75	A089_23 (pMMB207::*luxR*) mutant	“
A089_68	A089 Δ*luxS* mutant	“
A089_84	A089_68 (pMMB207::*luxS*) mutant	“
A089_92	A089_68 (pMMB207)	“
A102_56	A102 Δ*luxR* mutant	“
A102_78	A102_56 (pMMB207::*luxR*) mutant	“
A102_90	A102_56 (pMMB207)	“
A102_73	A102 Δ*luxS* mutant	“
A102_87	A102_73 (pMMB207::*luxS*) mutant	“
A102_94	A102_73 (pMMB207)	“
A102_pACYC	A102 (pACYC184)	[11]
A102_6.2	A102 (pACYC184::*aiiA*)	“
A102_99	A102_73 (pACYC::*luxS*)	This study
*E. coli* strains		
DH5α	E. *coli* used for transformation: λpir	Promega
S17-1	E. *coli* used for conjugation: λpir mob	[32]
Plasmids		
pDM4	Cm^r^ Kan^r^ SacBR; suicide vector	[28]
pMMB207	Cm^r^_,_*Ptac*, broad host range expression vector	[33]
pACYC184	Tet^r^, Cm^r^, broad host range expression vector	[34]
pGEM T-easy	Amp^r^ Kan^r^; TA cloning vector for sequencing	Promega

### Detection of *luxR* homologues by PCR

Primers luxRI-F2 and luxRI-R2 (Table 1) were designed based on *V. harveyi luxR* and *luxR* homologue sequences from other vibrios retrieved from GenBank. Genomic DNA was used as template. Genomic DNA was isolated from single colonies by inoculating them in 20 μl of double distilled H_2_O and boiling for 10 min. The samples where then chilled and centrifuged for 5 min at 16,000 *g* and 5 μl of the supernatant was used as template for the PCR. The primers and reagents for PCR were purchased from Roche Diagnostics (Barcelona, Spain). The conditions used for the PCR are described elsewhere [26]. A 636-bp fragment containing part of the *luxR* gene was obtained.

### Cloning and sequencing of *luxR* gene and its flanking DNA

The DNA sequence of the entire *luxR* gene of the two strains of *V. scophthalmi* together with the 5’- and 3’- flanking regions was obtained by inverted PCR [27]. To prepare template for the inverted PCR, genomic DNA was digested with the restriction enzyme *Hinc*II and the linear *Hinc*II fragments were circularized by ligation with T4 DNA ligase (Invitrogen). The ligated DNA molecules were used as template to amplify a DNA fragment on which the 5’- and 3’-ends of the *luxR* gene have been joined at a *Hinc*II site. To amplify this fragment, primers (LuxRI-R4 and LuxRI-F4, Table 1) were designed to polymerize DNA out from either end of the 636-bp fragment that contains part of the *luxR* gene. A single amplimer was generated and sequenced to identify the flanking ends of the *luxR* gene. Using this sequence data, primers (LuxR-1 and LuxR-2, Table 1) were designed to amplify the entire *luxR* gene plus the 5’- and 3’-flanking DNA (a total of 944 bp). This fragment was cloned and sequenced using the LuxR-1 and LuxR-2 primers. These sequences were submitted to the GenBank database under the accession number JN684209 and JN684210, for *V. scophthalmi* A089 and A102, respectively.

### Sequencing of DNA that flanks the *luxS* gene

The flanking regions of the previously sequenced *luxS* gene (accession number EF363481) were obtained as described above for *luxR*, except that the restriction enzyme *Dra*I and the primers LuxS-F6 and LuxS-R7 were used (Table 1).

### DNA sequencing

DNA sequencing was performed with the Big Dye Terminator Cycle Sequencing Ready Reaction Kit 3.1 (Applied Biosystems), according to the manufacturer’s instructions.

### Construction of Δ*luxR* and Δ*luxS* mutants by allelic exchange

In-frame deletions of the *luxR* and *luxS* genes were generated by allelic exchange as previously described [28]. Briefly, an altered allele for both the *luxR* and the *luxS* genes was created by overlap PCR that encodes the first 12 amino acids fused to the last 9 amino acids, for *luxR* and the first 9 amino acids fused to the last 9 amino acids for *luxS*. The PCR primers LuxR-A, LuxR-B and LuxR-C, LuxR-D (Table 1) were used to create the *luxR* mutant allele and primers LuxS-A, LuxS-B, LuxS-C, and LuxS-D (Table 1) were used to create the *luxS* mutant allele. Both alleles were cloned into the R6K-origin based suicide vector pDM4 creating pDM4-luxR-AD and pDM4-luxS-AD, respectively. These plasmids were transferred to the *V. scophthalmi* A089 and A102 parental strains by bacterial conjugation as stated below, generating the *V. scophthalmi* A089_23 and A102_56 mutant, which carry a *luxR* in-frame deletion, and the *V. scophthalmi* A089_68 and A102_73 mutants, which carry a *luxS* in-frame deletion.

### Construction of mutants over-expressing *luxR* and *luxS* genes

In order to determine the effect of over-expressing the *luxR* gene, the *luxR* and *luxS* genes were cloned into pMMB207 and fused to the *tac* promoter, which was induced using 0.5 mM IPTG. To clone into this vector, primers LuxR-G and LuxR-H were used for *luxR* and LuxS-PMMBF and LuxS-PMMBR for *luxS*. In order to tranfer the pMMB207 plasmid alone or the pMMB207 plasmid carrying the *luxS* or *luxR* genes to *V. scophthalmi luxR* and *luxS* null mutants, the plasmid constructions were electroporated into *E. coli* S17-1. The plasmids were later transferred to *V. scophthalmi* by bacterial conjugation as stated below.

### Complementation of *luxS* null mutant

Complementation of the A102_73 *luxS* mutant was performed by amplification of *luxS* gene with primers LuxS-AI and LuxS-BI (Table 1), followed by digestion with Bam*HI* and Sal*I* and ligation to the pACYC plasmid digested with the same strains (Table 3). The pACYC plasmid carrying the *luxS* gene was then electroporated into *E. coli* S17-1 (Table 3) and the transformants selected using 20 μg/ml chloramphenicol LB plates. This plasmid was later transferred to *V. scophthalmi* by bacterial conjugation and selected in TCBS with 5 μg/ml as stated below.

### Bacterial conjugation

Plasmids pMMB207, pMMB207*::luxR*, pMMB207*::luxS* and pACYC*::luxS* cloned into *E. coli* S17-1 were mobilized into *V. scophthalmi* by bacterial conjugation. Briefly, the *E. coli* S17-1 carrying the corresponding plasmid and the *V. scophthalmi* receptor strain were grown to mid-logarithmic growth phase. A total of 0.5 ml of the *E. coli* culture was pelleted in a microfuge, the supernatant was removed, and the cells were mixed with 1 ml of *V. scophthalmi*. The cell mixture was centrifuged and suspended in 50 μl of TSB2. The 50 μl were spotted onto a TSA2 plate and incubated at 30°C for 24 h. Following incubation, the bacterial cells were resuspended in TSB2 and serial dilutions were plated onto TCBS medium (Oxoid) containing 5 μg/ml chloramphenicol to select for the *V. scophthalmi* containing the plasmids.

In order to construct the *V. scophthalmi luxR* and *luxS* null mutants, the *E. coli* S17-1 strains carrying either pDM4-luxR-AD and pDM4-luxS-AD were mated with *V. scophthalmi* A089 and A102 wild type strains. The selection for the strains carrying the suicide plasmid was performed in TCBS containing 5 μg/ml chloramphenicol as stated above. Afterwards, the null mutants were further selected after induction of sacBR in TSB2 agar plates supplemented with 5% sucrose. The in-frame deletions were confirmed by sequencing a PCR-amplified DNA fragment containing each mutation.

### Phenotypic assays

#### Growth rate

The effect of the mutations on the growth rate of these bacteria was analysed. Briefly, ON cultures were prepared on TSB2 and diluted to an initial density of approximately 0.01 and incubated for 10 h at 30°C with continuous agitation. Bacterial growth was estimated from OD readings at 600 nm taken at different intervals.

#### Protease activity

Extracellular protease activity was evaluated both qualitatively and quantitatively. For qualitative assay the parental as well as the mutant strains were streaked onto TSA2 and MA supplemented with 1%, 1.5% or 2% skimmed milk and incubated for a maximum of 48 h. The presence of a casein degradation halus was considered a positive result. The quantitative assay was performed as previously described using the azocasein assay as previously described [29], using O/N supernatants of the strains to be tested.

#### Biofilm formation

Biofilm formation was evaluated using 96-well polystyrene cell-culture treated microtiter plates after 48 h incubation using the crystal violet staining method, as previously described [30]. Briefly, O/N cultures of the corresponding strain to be tested were diluted into fresh TSB2 or MB media to get approximately an optical density of 0.01 OD_600 nm_ units. A total of 200 μl were dispensed in each well and incubated statically in a wet chamber for 48 h at 30°C. A minimum of four replicates in three independent assays were measured.

#### Motility

MA and TSA2 swimming plates containing 0.25% agar were used to assess the effect of LuxS and LuxR in motility. An overnight culture of the corresponding strain to be analysed was diluted 1:100 and a drop containing 10 μl of the sample was inoculated in the middle of the plate and the movement of the strains was monitored up to 48 h by measuring the diameter reached by the bacteria.

#### Detection of siderophores

The chrome azure assay (CAS) was used to detect the production of siderophores in both the mutants and wild type strains, as described in [31] with minor modifications. Briefly, the nutrient medium used for the growth of the bacteria was TSA supplemented with 0.5% NaCl. Additionally, the ability of these strains to grow on iron depleted media was assessed using MA and TSA2 plates containing 0.2 mM ethylenediamine di(o-hydroxyphenylacetic acid) (EDDA) chelating agent.

### Membrane protein profiling by mass spectrometry

Membrane proteins from the mutants and wild type strains were extracted from 500 ml ON cultures. Briefly, the cultures were centrifuged for 10 min at 16,000 *g* and washed with PBS. The cells were suspended in 10 ml Tris 50 mM pH 8.0 and the suspension was frozen at −80°C. Successive rounds of freezing and thawing were performed. The suspension was then centrifuged for 2 min at 16,000 *g*. The supernatant was centrifuged at 16,000 *g* for 1 hour at 4°C and the pellet enriched in membrane proteins was suspended in 10 μl of 50% acetonitrile-2.5% trifluoroacetic acid. One microliter of the supernatant was placed onto a spot of a ground steel plate and air dried at room temperature. Each sample was overlaid with 1 μl of matrix solution (saturated solution of α-cyno-4-hydroxy-cinnamic acid in 50% acetonitrile-2.5% trifluoroacetic acid) and air dried at room temperature.

Measurements were performed on an Autoflex III MALDI-TOF/TOF mass spectrometer (Bruker Daltonics, Leipzig, Germany) equipped with a 200-Hz Smartbeam laser. Spectra were recorded in the linear, positive mode at a laser frequency of 200 Hz within a mass range from 2,000 to 20,000 Da. The IS1 voltage was 20 kV, the IS2 voltage was maintained at 18.7 kV, the lens voltage was 6.50 kV, and the extraction delay time was 120 ns.

For each spectrum approximately 500 shots from different positions of the target spot were collected and analyzed. The spectra were calibrated externally using the Bruker Bacterial Test Standard (*Escherichia coli* extract including the additional proteins RNase A and myoglobin). Calibration masses were as follows: RL29 3637.8 Da; RS32, 5096.8 Da; RS34, 5381.4 Da; RL33meth, 6255.4 Da; RL29, 7274.5 Da; RS19, 10300.1 Da; RNase A, 13683.2 Da; myoglobin, 16952.3 Da). The analyses were performed in triplicate.

## Competing interests

The authors declare that they have no competing interests.

## Authors’ contributions

CGA participated in the design, acquisition of data and wrote the manuscript; SMR participated in the acquisition and analysis of data; DLM has participated in the design of the study and has helped writing the manuscript; ARB participated in the design of the study and revision of the manuscript. All authors have read and approved the final version of the manuscript.
